# Heterotrophy in the earliest gut: a single-cell view of heterotrophic carbon and nitrogen assimilation in sponge-microbe symbioses

**DOI:** 10.1038/s41396-020-0706-3

**Published:** 2020-06-29

**Authors:** Laura Rix, Marta Ribes, Rafel Coma, Martin T. Jahn, Jasper M. de Goeij, Dick van Oevelen, Stéphane Escrig, Anders Meibom, Ute Hentschel

**Affiliations:** 1 RD3 Marine Symbioses, GEOMAR Helmholtz Centre for Ocean Research Kiel, Düsternbrooker Weg 20, 24105 Kiel, Germany; 2 School of Biological Sciences, University of Queensland, St. Lucia, QLD 4072 Australia; 3 Department of Marine Biology and Oceanography, Institute of Marine Science, ICM-CSIC, Barcelona, Spain; 4 Department of Marine Ecology, Centre for Advanced Studies, CEAB-CSIC, Blanes, Spain; 5 Department of Freshwater and Marine Ecology, Institute for Biodiversity and Ecosystem Dynamics, University of Amsterdam, PO Box 94248, 1090 GE Amsterdam, The Netherlands; 6 Department of Estuarine and Delta Systems, NIOZ Royal Netherlands Institute for Sea Research, and Utrecht University, PO Box 140, 4400 AC Yerseke, The Netherlands; 7 Laboratory for Biological Geochemistry, School of Architecture Civil and Environmental Engineering, Ecole Polytechnique Fédérale de Lausanne (EPFL), CH-1015 Lausanne, Switzerland; 8 Center for Advanced Surface Analysis, Institute of Earth Sciences, University of Lausanne, CH-1015 Lausanne, Switzerland; 9 Christian-Albrechts-University of Kiel (CAU), Kiel, Germany

**Keywords:** Symbiosis, Stable isotope analysis, Animal physiology

## Abstract

Sponges are the oldest known extant animal-microbe symbiosis. These ubiquitous benthic animals play an important role in marine ecosystems in the cycling of dissolved organic matter (DOM), the largest source of organic matter on Earth. The conventional view on DOM cycling through microbial processing has been challenged by the interaction between this efficient filter-feeding host and its diverse and abundant microbiome. Here we quantify, for the first time, the role of host cells and microbial symbionts in sponge heterotrophy. We combined stable isotope probing and nanoscale secondary ion mass spectrometry to compare the processing of different sources of DOM (glucose, amino acids, algal-produced) and particulate organic matter (POM) by a high-microbial abundance (HMA) and low-microbial abundance (LMA) sponge with single-cell resolution. Contrary to common notion, we found that both microbial symbionts and host choanocyte (i.e. filter) cells and were active in DOM uptake. Although all DOM sources were assimilated by both sponges, higher microbial biomass in the HMA sponge corresponded to an increased capacity to process a greater variety of dissolved compounds. Nevertheless, in situ feeding data demonstrated that DOM was the primary carbon source for both the LMA and HMA sponge, accounting for ~90% of their heterotrophic diets. Microbes accounted for the majority (65–87%) of DOM assimilated by the HMA sponge (and ~60% of its total heterotrophic diet) but <5% in the LMA sponge. We propose that the evolutionary success of sponges is due to their different strategies to exploit the vast reservoir of DOM in the ocean.

## Introduction

As the oldest extant animal phyla, sponges (phylum Porifera) have thrived on Earth for more than 600 million years [1, 2]. These sessile, filter-feeding invertebrates are ubiquitous from the tropics to the poles and from freshwater mountain lakes to the deep-sea floor thousands of meters deep [3]. In the many ecosystems where they are abundant (e.g., coral reefs and sponge grounds), sponges play a major role in nutrient cycles [4, 5], due to both their unrivaled capacity to filter seawater [6, 7] and their association with diverse and abundant microbial symbionts [7, 8]. Sponges and their symbionts have evolved multiple nutritional strategies to cope with the vastly different environments they occupy, including photosynthesis on shallow, oligotrophic coral reefs [9], chemosynthesis at hydro-carbon seeps [10], and even carnivory in the food-limited deep-sea [11]; however, the majority of sponges are chiefly heterotrophic filter-feeders that capture food using specialized feeding cells (choanocytes). Sponge symbionts play a well-known role in sponge autotrophy, yet, their role in sponge heterotrophy remains enigmatic.

The largest potential source of heterotrophic food in the oceans is dissolved organic matter (DOM) [12], but this food source is largely inaccessible to most marine animals. Instead, DOM is primarily utilized by heterotrophic microbes who recycle as much as 50% total marine productivity through the microbial loop [13]. Sponges have long been hypothesized to utilize DOM [14–16], but only recently has there been growing consensus that DOM accounts for a significant proportion (up to 97%) of the sponge diet [17–24]. Sponge assimilated DOM is subsequently made available as a food source for other marine animals through consumption of sponge-generated detritus through a pathway called the sponge loop [25] and predation on sponge biomass [26]. Within coral reefs, sponge recycling of DOM into the classical food chain is estimated to be on the same order of magnitude as gross primary production rates of the entire ecosystem [25]. This capacity for DOM processing by sponges is remarkable given that although other marine invertebrates can assimilate limited amounts of DOM [27–29], apart from larval life stages [30], no other multicellular marine animal is known to use DOM to meet the vast majority (>90%) of its metabolic demand [17, 20], leading to the widespread postulation that its assimilation is facilitated by microbial symbionts [6, 15].

The microbial communities associated with sponges are exceptionally diverse [8], forming stable and species-specific associations that can account for as much as 35% of the total sponge biomass [31, 32]. Intriguingly, these symbionts are not evenly distributed across species, but can be categorized into two distinct groups with high-microbial abundance (HMA) sponges harboring densities of microbes 2–4 orders of magnitude higher than low-microbial abundance (LMA) sponges [31, 33, 34]. Evolutionary rationales for this dichotomy are unknown, but HMA and LMA sponges further differ in the type and diversity of their microbial symbionts [34], host physiology [6, 35], and nutrient processing [22, 36], including their capacity to process DOM. Since microbes are assumed to play an important role in mediating sponge DOM uptake, it is hypothesized that HMA sponges are better adapted for DOM uptake than LMA sponges [6, 15]. However, recent research has produced conflicting results; while some studies have found evidence to support this hypothesis [20, 23], others have found similar DOM uptake rates in both HMA and LMA sponges [5, 17, 18, 22]. Evidence from compound-specific stable isotope probing (SIP) indicates that both host sponge cells and bacterial symbionts may be active in DOM assimilation [37–39] and direct uptake by host cells has been detected by NanoSIMS in a photosynthetic sponge [40]. However, the quantitative contribution of host cells or microbial symbionts to DOM assimilation remains unknown.

**Fig. 1:**
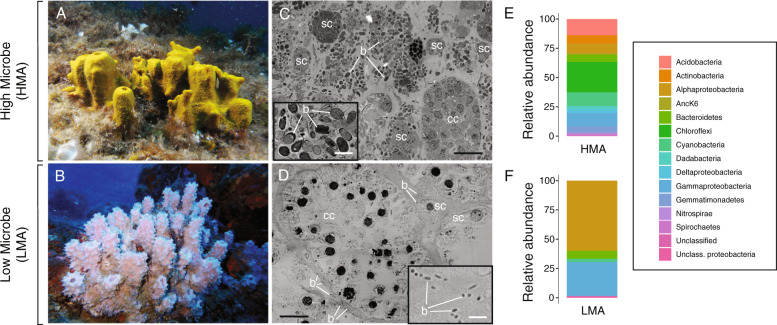
Comparative morphology and microbial diversity of the HMA sponge *Aplysina aerophoba* and LMA sponge *Dysidea avara*. **a, b** In situ photographs depicting the similar massive growth form of the two sponge species consisting of clumps of individual “chimneys” each containing a single osculum. **c**, **d** Scanning electron micrographs of the internal sponge morphology. The mesohyl of the HMA sponge *A. aerophoba* is more densely packed with bacteria and sponge cells and contains smaller choanocyte chambers compared with the LMA sponge *D. avara*, which has large choanocyte chambers and less dense tissue with sparse bacteria. Bacterial symbionts are larger and more abundant in the HMA sponge *A. aerophoba*. **e**, **f** Microbial community composition of the two sponges based on 16S rRNA gene data (*n* = 6), showing that the HMA sponge *A. aerophoba* has a more diverse microbial community (Shannon index; *P* < 0.05). m mesohyl, cc choanocyte chamber, sc sponge cell, b bacterial symbionts. Scale bars: **c, d** 10 µm, insets 1 µm.

To quantify host and symbiont contributions to sponge DOM uptake, we performed SIP experiments to compare the processing of three dissolved food sources (glucose, amino acids, and algal DOM) and one particulate food source (heterotrophic bacteria) in the HMA sponge *Aplysina aerophoba* (Fig. 1a) and LMA sponge *Dysidea avara* (Fig. 1b). These two species typify the HMA–LMA dichotomy with *A. aerophoba* hosting a more abundant (Fig. 1c, d) and more diverse (Fig. 1e, f) microbial community than *D. avara*. Host and symbiont contributions to the uptake and assimilation of DOM in the two species were quantified through stable isotope analysis of (i) bulk sponge tissue, (ii) separated sponge and microbial cell fractions, and (iii) single cells with subcellular resolution NanoSIMS imaging. SIP was complemented with in situ measurements of the natural sponge diet to determine the total heterotrophic contribution of sponge symbionts to the sponge diet.

## Materials and methods

### Organism collection

Specimens of *Aplysina aerophoba* and *Dysidea avara* (*n* = 20) were collected by SCUBA from the coast of Girona, Spain (42° 06′ 55″ N, 3° 10′ 8″ E) at depths of 3–15 m during April and May 2017. Sponges were transferred to the aquaria facilities at the Institute of Marine Sciences (ICM-CSIC) in Barcelona and maintained in individual 6 L aquaria supplied with fresh flowing seawater at a rate of ~30 L h^−1^. Each sponge individual was divided into five fragments of a similar size, each with a single fully functional osculum, and attached to PVC plates. Sponges were acclimated for 5 days and only healthy, actively pumping individuals were used in experiments.

### Stable isotope pulse-chase labeling experiments

**Fig. 2:**
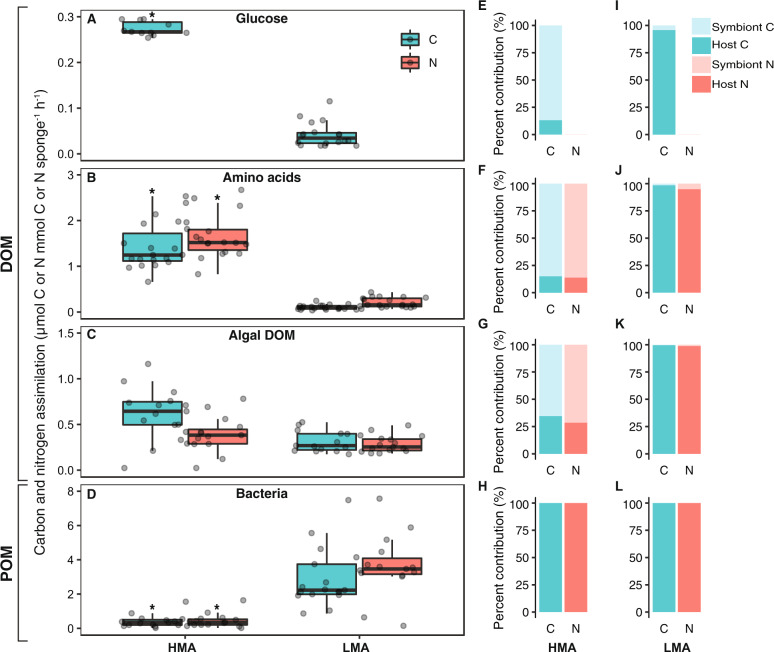
Bulk tissue assimilation of the four food sources and contribution of host and symbiont cells to total uptake. Rates of carbon (C) and nitrogen (N) assimilation of the four food sources **a** glucose, **b** amino acids, **c** algal DOM, and **d** bacteria into the bulk tissue of the HMA sponge *A. aerophoba* and LMA sponge *D. avara*. Rates presented as µmol C or N assimilated per mmol C or N sponge tissue per hour. Significant differences in C or N assimilation between the two sponge species are marked (*) for each food source (significance level *P*_(perm)_ < 0.05). Boxplots depict the 25th percentile, median, and 75th percentile overlaid with the raw data points (*n* = 20). (**e**–**l**) Percent contribution of host sponge cells (dark colors) and symbiont microbes (light colors) to total assimilation of the four food sources for both carbon (green) and nitrogen (orange) as determined by isotopic enrichment of separated cell fractions. .

Stable isotope pulse-chase experiments were conducted to test for the assimilation of three dissolved (^13^C-glucose, ^13^C- and ^15^N-amino acids, ^13^C- and ^15^N-algal DOM) and one particulate food source (^13^C- and ^15^N-labeled bacteria). Details of the preparation of the four food sources are described in the Supplementary Methods. Food sources were added to individual 6 L aquaria (1 sponge fragment per aquaria) at a concentration of ~80 µmol L^−1^ C, approximately equivalent to the background concentrations of DOC in the surrounding seawater (60–120 µmol L^−1^ C). Total amounts C and N added and enrichment of the four food sources is listed in Table S1. Small aquaria pumps ensured water circulation during the 3 h pulse incubation and aquaria were kept in a water bath of free-flowing seawater to ensure maintenance of ambient seawater temperature. Sponges were sampled at 5 time points: three pulse time points (0.5, 1, and 3 h) after which all remaining sponges were rinsed in label-free seawater and transferred without air exposure to label-free aquaria and sampled at two chase points (6 and 9 h). Each time point consisted of four replicates with one fragment from each individual used in each time point (total of *n* = 20 replicates per treatment). Control samples were collected before (*n* = 4) and after (*n* = 4) the experiments. The pulse-chase design was conducted to test for potential translocation of C and N from symbiont to host cells. However, since no significant differences in host cell enrichment were detected in the chase samples (Fig. S1, details in Supplementary Results), only samples from the 3-h pulse time point were measured for NanoSIMS analysis. In addition, we found no significant difference in bulk uptake rates between time points (Table S2) and therefore time points were pooled to generate Fig. 2. Any sponges that ceased pumping during the experiment were excluded from analyses.

Sponge tissue samples were collected for (1) stable isotope analysis of bulk sponge tissue, (2) stable isotope analysis of separated sponge and microbial cell fractions, and (3) SEM and NanoSIMS. Samples for SEM and NanoSIMS were sampled with a 2-mm tissue biopsy punch and immediately fixed in 4% paraformaldehyde in PBS buffer for 12 h at 4 °C and then transferred to PBS and stored at 4 °C until further processing. Samples for isotope analysis of the bulk sponge tissue (~1 cm^3^) were rinsed in filtered seawater followed by a brief rinse with MQ to remove excess salt and frozen at −80 °C. The remainder of the sponge tissue was cut into small pieces and placed in ice-cold calcium- and magnesium-free artificial seawater with 10% EDTA at 4 °C for separation into sponge cell and microbial cell fractions (details in Supplementary Methods).

### Stable isotope analysis and calculations of food uptake rates

Bulk tissue samples were lyophilized, homogenized, and sub-samples weighed into silver (C) and tin (N) cups for stable isotope analysis of δ^13^C and δ^15^N. Samples for δ^13^C were decalcified with 0.4 M HCl to obtain the organic carbon content. Separated cell fractions were lyophilized and weighed into tin cups for simultaneous δ^13^C and δ^15^N as test samples indicated that acidification was not required. Isotope ratios and C/N content were simultaneously measured using a Thermo FlashEA 1112 elemental analyzer coupled to a Delta V isotope ratio mass spectrometer. Carbon and nitrogen stable isotope ratios are expressed in standard delta notation as

1 $$\delta ^{13}{\mathrm{C}}\;{\mathrm{or}}\;\delta ^{15}{\mathrm{N}}(\permil) = \left( {\frac{{R_{{\mathrm{sample}}}}}{{R_{{\mathrm{ref}}}}} - 1} \right) \times {\mathrm{1000}},$$

where *R* is the ratio of ^13^C/^12^C or ^15^N/^14^N in the sample or reference material: Vienna Pee Dee Belemnite for C (*R*_ref_ = 0.01118) and atmospheric nitrogen for N (*R*_ref_ = 0.00368 N). Bulk tissue uptake rates presented in Fig. 2a–d were calculated as per ref. [37] (see Supplementary Methods).

### Calculations host and symbiont percent contribution to DOM uptake

Sponge and microbial cell fractions were separated by centrifugation using methods adapted from Wehrl et al. [41] and Freeman et al. [42] (details in Supplementary Methods). Briefly, sponge tissue was gently homogenized by mortar and pestle and the resulting dissociated cells were separated by centrifugation into a sponge cell fraction and microbial cell fraction. Sub-samples were collected pre-separation from the initial homogenate to determine the total number of sponge and microbial cells present in the sponge tissue and post-separation to determine the purity of the resulting sponge and microbial cell fractions. Cell numbers were counted using DAPI staining (see Supplementary Methods for details). All 3 h controls and samples were counted (*N* = 56). Purity of cell fractions was >99% for the microbial cell fractions and >85% for the sponge cell fractions (Table S3).

To determine C and N contents of the sponge and microbial cells for biomass calculations, known volumes of the separated cell fractions were filtered onto a pre-combusted GF/F filter for elemental CN analysis (*n* = 16). Cell counts indicated that >99.5% of the microbial cells were effectively captured on the filters. The C and N content of the filters was divided by the number of cells filtered to calculate the mean C and N content per sponge and microbial cell for each of the two species. The mean C and N contents were multiplied by the total number of sponge and microbial cells in the initial homogenate (pre-separation) to calculate the percent biomass of sponge and microbial C and N in the two sponge species.

The C/N biomass of sponge and microbial cells and the ^13^C and ^15^N enrichment of the two cell fractions (determined independently by isotope analysis of separated cell fractions and NanoSIMS) were used to calculate the percent contributions of host cells and microbial symbionts to the total uptake rates of the three dissolved food sources shown in Fig. 2 and Table S4. It was assumed that host sponge cells were responsible for 100% of the uptake of the particulate food source (bacteria) based on known mechanisms of sponge feeding [43–45].

### Scanning electron microscopy (SEM) and NanoSIMS

Fixed tissue samples from the pulse-chase experiment were dehydrated in a series of ethanol and embedded in LR-white for correlative SEM and NanoSIMS. Ultrathin tissue sections (~120 nm) were mounted onto silicon wafers and stained with uranyl acetate and lead citrate before imaging on a Zeiss Gemini 500 field emission variable pressure SEM equipped with energy selective backscatter detector and secondary ion detector at 5 kv. Images with ~50 × 50 µm field-of-view containing cellular structures of interest were mapped for subsequent NanoSIMS analysis.

To examine ^13^C and ^15^N enrichment in the two sponges at single-cell resolution, the selected areas mapped by SEM were analyzed for ^13^C and ^15^N enrichment using a NanoSIMS 50 L ion probe (CAMECA). SEM sections on silicon wafers were gold-coated and bombarded with a 16 keV primary Cs+ ion beam focused to a spot size of ~120 nm. Raster scans (30 × 30 µm, 256 × 256 pixels) of the areas of interest were performed with a beam dwell time of 5 ms per pixel and repeated ten times. The secondary ions ^12^C_2_^−^ (mass 24), ^13^C^12^C^−^ (mass 25), ^12^C^14^N^−^ (mass 26), and ^12^C^15^N^−^ (mass 27) were simultaneously collected using electron multipliers at a mass resolution (*M*/Δ*M*) of 9000. Unlabeled sponges were measured twice daily as controls.

NanoSIMS data were processed using the ImageJ plugin OpenMIMS in Fiji (National Resource for Imaging Mass Spectrometry, https://github.com/BWHCNI/OpenMIMS/wiki). Maps of ^13^C/^12^C and ^15^N/^14^N enrichment were obtained by taking the ratio of the drift corrected and stacked ^13^C^12^C^−^ and ^12^C_2_^−^ or ^12^C^15^N^−^ and ^12^C^14^N^−^ images, respectively. Quantification of isotope ratios in the different cell types was achieved by manually drawing regions of interest (ROIs) on the ^12^C^14^N-image using the corresponding SEM maps for reference. These SEM images were also used to identify the cell types present in the NanoSIMS images. The analysis focused on three main cell types (1) symbiont bacteria (0.2–2 µm), (2) host choanocyte cells (3–10 µm), and (3) all other host cells in the sponge mesophyll and pinacoderm, including ameobocytes, archeocytes, spherulous cells, and pinacocytes (7–30 µm). Hotspots of enrichment in the host choanocyte cells were extracted from the isotope ratio images separately. A total of 16 samples from the 3-h pulse time point were analyzed: two samples per species from each treatment (apart from the glucose treatment where enrichment levels were low and only one sample was measured) and two controls (one for each species). Approximately 4–8 images were obtained per sample in order to capture a minimum of *n* = 20 for each cell or ROI type, and in total 4354 ROIs were defined (Table S5). ROIs were considered enriched if their δ^13^C or δ^15^N values were more than two standard deviations above the average of a similar set of ROIs from the control samples. Delta values (‰) were calculated as above replacing the reference values with the measured ^13^C/^12^C and ^15^N/^14^N ratios of the control samples (measured as ^13^C^12^C^−^/^12^C^12^C^−^ and ^15^N^12^C^−^/^14^N^12^C^−^, respectively).

### In situ measurements of the natural sponge diet

The natural diet of the two sponge species were measured *in situ* by SCUBA using the InEx VacuSIP technique (see Morganti et al. [46] for full methodological details). Sampling for *A. aerophoba* was conducted in May to June 2017 off the coast of Girona, Spain (42° 03′ 34″ N 3° 12′ 51″ E) between 5 and 15 m water depth. Data for *D. avara* was taken from Morganti et al. [22]. Briefly, net fluxes were determined by measuring concentration differences between the water inhaled (In) and exhaled (Ex) by the sponge. Exhalant water was sampled directly from the sponge osculum while inhalant water was measured a few cm away using a custom setup that used vacuum pressure to draw in water. The sampling rate (<1 mL min^−1^) was kept sufficiently below the sponge pumping rate to avoid contamination of the Ex sample with ambient of water. Samples were taken for dissolved organic carbon (DOC) and pico- and nanoplankton as the primary particulate organic carbon (POC) diet component (sampling details in Supplementary Methods). Sponge pumping rates were calculated using the dye front speed method as described by Morganti et al. [7]. Uptake rates (C_flux_) of DOC and POC were calculated as

6 $$ C_{{\mathrm{flux}}} = \Delta C_{{\mathrm{in - ex}}} \times P_{{\mathrm{sponge}}},$$

where Δ*C*_in-ex_ is the net flux of DOC or POC (μmol C L^−1^) and *P*_sponge_ is the sponge pumping rate normalized to sponge volume (ml min^−1^ cm^−3^). Total organic carbon (TOC) uptake rates were calculated as: *C*_TOC_ = *C*_POC_ + *C*_DOC_ (μmol C min^−1^ cm^−3^). Symbiont contributions to *C*_DOC_ and *C*_TOC_ uptake were calculated using the percent contribution of bacterial symbionts to algal DOC uptake as algal DOM is most representative of the natural DOM pool available in situ.

### Statistical analyses

Statistical analyses were conducted in PRIMERv7 [47] with the permutational analysis of variance+ (PERMANOVA) add-on [48]. Univariate PERMANOVAs were used to test for significant differences between groups. Dissimilarity matrices constructed using Euclidean distance and the *P*_(perm)_ value was based on 9999 permutations. Type III (partial) sums of squares were used to account for the unbalanced design of the NanoSIMS data. Post hoc comparisons were conducted when significant factor effects were found. Results were considered significant at the level *P*_(perm)_ < 0.05. Statistical outputs are summarized in Tables S2 and S6–S8.

## Results

### Differential processing of dissolved and particulate food by the high- and low-microbial abundance sponges

SIP experiments showed that all four food sources (glucose, amino acids, algal DOM, and bacteria) were assimilated into the bulk tissue of both the HMA sponge *A. aerophoba* and LMA sponge *D. avara*, but they exhibited significant differences in assimilation rates (Fig. 2a–d). The HMA sponge showed highest uptake of amino acids (significantly compared with all other food sources; Table S6), but all dissolved food sources assimilated at a rate similar or higher than the particulate food source (bacteria) (Fig. 2a–d). By contrast, the LMA sponge showed highest assimilation of bacteria, which was assimilated at a significantly higher rate (by at least ten times) than for any of the dissolved food sources (*P*_perm_ < 0.05 in all cases, Table S6). Of the DOM sources, glucose was taken up at a significantly lower rate than the other food sources (*P*_perm_ < 0.05 for all comparisons), particularly by the LMA sponge which had glucose uptake rates seven 7 lower than for algal DOM and 70 times lower than for bacteria (Fig. 2c–f). When comparing the two species, the HMA sponge took up glucose and amino acids at a significantly higher rate (6 and 12 times higher, respectively) than the LMA sponge (both *P*_perm_ < 0.05, Table S7), but there was no significant differences between the two sponges in the uptake rates of algal DOM, the most representative natural DOM source. For the particulate bacteria food source, the HMA sponges showed significantly (eight times) lower assimilation rates than the LMA sponge (*P*_perm_ < 0.05, Table S7).

### Single-cell analysis reveals DOM is incorporated by both host and symbiont cell

**Fig. 3:**
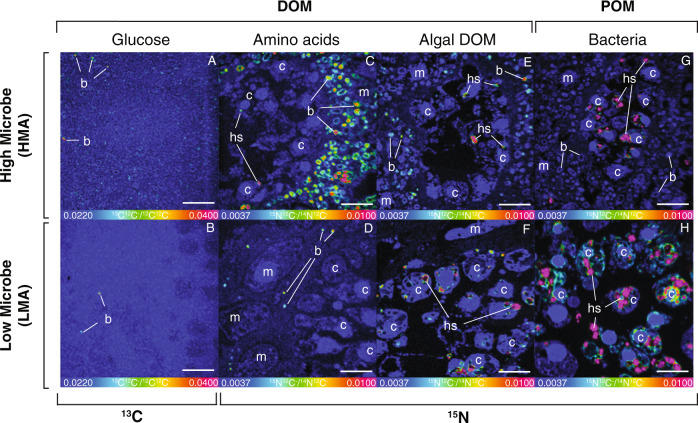
NanoSIMS visualization of the relative enrichment in ^13^C or ^15^N in sponge host and symbiont cells. The measured ratios (i.e., ^13^C^12^C^−^/^12^C^12^C^−^ and ^15^N^12^C^−^/^14^N^12^C^−^, respectively) are shown with a rainbow color-scale (hue saturation intensity) ranging from blue (natural abundance) to pink (most enriched regions) in *A. aerophoba* (HMA) and *D. avara* (LMA) tissue after the 3-h isotopic pulse with the four different ^13^C and ^15^N-labeled food sources: **a, b** glucose, **c, d** amino acids, **e, f** algal DOM, and **g, h** bacteria. Note the different *y*-axis scale for the bacterial food source (**g, h**). DOM dissolved organic matter, POM particulate organic matter, c host choanocyte cell, hs hotspot of enrichment in host choanocyte cell, m host mesophyll cell, b symbiont bacteria. Scale bars are 5 µm.

**Fig. 4:**
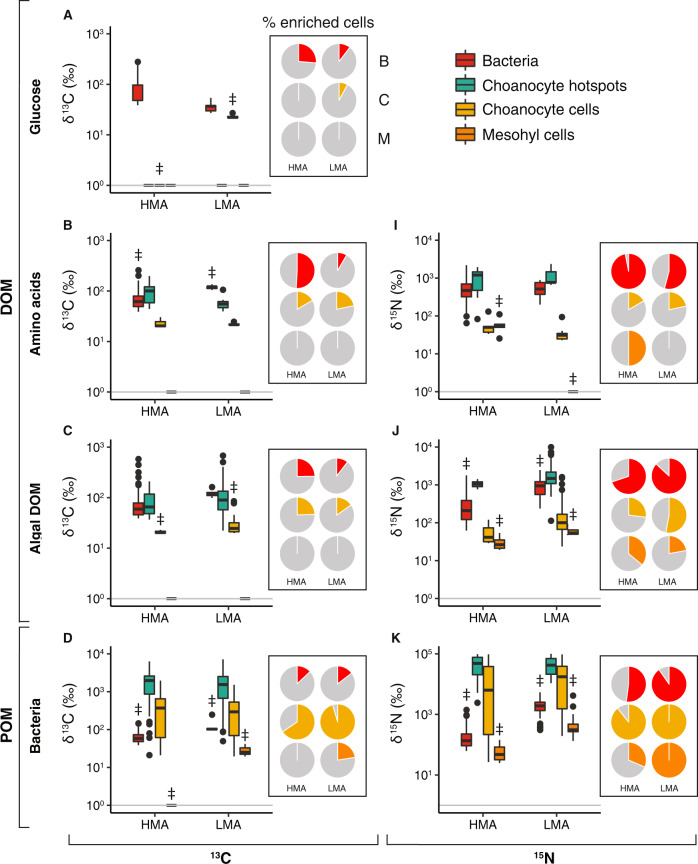
Single-cell quantification of above-background ^13^C and ^15^N enrichment in the HMA sponge *A. aerophoba* and LMA sponge *D. avara*. Four regions of interest (ROIs) were analyzed by NanoSIMS: (1) symbiont bacteria, (2) hotspots of enrichment in host choanocyte cells, (3) host choanocyte cells, and (4) host mesohyl cells. Above-background enrichment (‰) of δ^13^C (**a**–**d**) and δ^15^N (**e**–**g**) for each ROI type after the 3-h isotopic pulse with the four labeled food sources. A minimum of *n* = 20 ROIs were analyzed for each ROI type. Note the different log10 *y*-axis scales. Gray horizontal lines depict background enrichment of control samples. ^‡^ denotes significant difference in individual ROI types between the HMA and LMA sponge (*P*_(perm)_ < 0.05). Pie charts depict the proportion of ROIs enriched in ^13^C or ^15^N for each of the three cell types of interest: B symbiont bacteria, C choanocyte cells, M mesohyl cells.

Significant isotopic enrichment was found in all sponge cell and bacterial cell fractions for all treatments after cell separation (Fig. S1) and this was supported by NanoSIMS at single-cell level (Fig. 3). For both the HMA and LMA sponge, the NanoSIMS images showed clear incorporation of ^13^C and ^15^N from the dissolved food sources into both host and symbiont cells (Fig. 3a–f). The majority (64–97%) of microbial cells in both species showed uptake of amino acids and algal DOM and hotspots of isotopic enrichment for these DOM sources could also be seen in host cells. In congruence with the bulk tissue uptake rates (Fig. 2a), incorporation of glucose into sponge and symbiont cells was low; ^13^C enrichment was detected in fewer than 26 and 7% of microbes in the HMA (Fig. 3a) and LMA (Fig. 3b) sponge, respectively, and only at the lowest detectable level in host cells (Figs. 4a, e and S1). For all food sources, both dissolved and particulate, hotspots of enrichment in host cells were almost exclusively observed in the choanocyte cells, i.e., the filtering cells that form the choanocyte chambers where food is captured by the host (Fig. 3). Enrichment in the mesohyl (i.e., interstitial space between the inner choanoderm and the outer pinacoderm) was less frequently detected in amoebocyte cells, and very rarely in other mesohyl or pinacoderm cells, and only when there was already high uptake in a large proportion of the choanocyte cells or microbial cells in the case of the HMA sponge (Fig. 4), suggesting mesohyl and pinacoderm cells were not the primary initial uptake sites.

The NanoSIMS data demonstrated clear differences in food processing strategies between the HMA and LMA sponge. These differences were largely driven by the disparity in microbial abundances in the two species rather than variations in cellular uptake rates between the two sponge species; this could be seen qualitatively directly in the NanoSIMS images (Fig. 3), and quantitatively after image processing (Fig. 4). Above-background enrichment of ^13^C and ^15^N (measured in parts-per-thousand, ‰) in host cells and symbiont bacteria were overall very similar in the HMA and LMA sponges with few significant differences detected in the specific enrichment of choanocyte cells or choanocyte hotspots and bacteria between the two sponges (Fig. 4 and Table S8). In general, a higher proportion of microbial cells were enriched in the HMA sponge while a higher proportion of choanocyte cells tended to be enriched in the LMA sponge, but these differences were generally small (Fig. 4, pie chart panels). The exception being when fed with bacteria (POM), which resulted in a higher proportion of enriched microbial symbiont cells in the LMA sponge demonstrating rapid recycling of host-processed particulate C and N by the symbiont microbes (details in Supplementary Results). Consequently, the differences in the overall uptake rates of the four food sources (Fig. 2a–d) are likely driven more by the substantial differences in host and symbiont biomass in the HMA and LMA sponge (Fig. 3) rather than differences in single-cell activities of the host and symbiont cells in the HMA versus LMA sponge (Fig. 4).

These differences in symbiont biomass led to substantial differences in the relative symbiont contributions to DOM uptake by the HMA and LMA sponge (Fig. 2e–l). Across the three DOM sources, incorporation into microbial cells accounted for 65–87% of the dissolved C and 72–86% of the dissolved N assimilated by the HMA sponge *A. aerophoba*. By contrast, <5% of the dissolved C and N assimilated by the LMA sponge *D. avara* was incorporated into microbial cells with the majority of DOM being assimilated by host sponge cells (up to >99% for the algal DOM). Quantitative NanoSIMS imaging produced remarkably similar results (Table S4), consistently showing that while microbial symbionts accounted for the majority of DOM uptake in the HMA sponge, host sponge cells were responsible for most of the DOM taken up by the LMA sponge. Although we did not detect translocation from symbiont to host cells, we did observe the HMA sponge phagocytosing its symbionts (Fig. S2, Supplementary Results).

### Microbial contributions to total sponge heterotrophic diet are higher in the HMA sponge

**Fig. 5:**
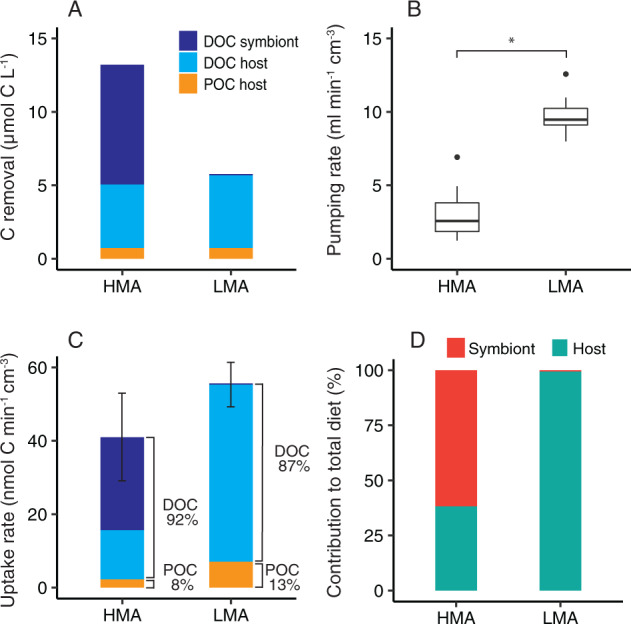
Natural diets of the HMA sponge *Aplysina aerophoba* and LMA sponge *Dysidea avara*. **a** Carbon removal rate (DOC + POC) determined from in situ measurements of the seawater inhaled and exhaled by the sponges using the VacuSIP technique. **b** Pumping rates of HMA and LMA sponges. **c** Total uptake rate with carbon removal normalized to pumping rate. **d** Percent contribution of host sponge cells and symbiont microbes to total heterotrophic diet. Symbiont microbes accounted for 58.6% of the total C (DOC + POC) taken up by the HMA sponge, but <1% in the LMA sponge. *A. aerophoba* data *n* = 10. *D. avara* feeding data from [22] (*n* = 6) and pumping data from [7] (*n* = 10). DOC dissolved organic carbon, POC particulate organic carbon. ^*^indicates significant difference between the HMA and LMA sponge (*P*_(perm)_ < 0_._05).

Despite significant differences in DOM uptake rates between the two sponges in the isotope tracer experiments, in which food was supplied in excess of natural concentrations (Fig. 2a–d), we found that the natural diets of the two sponges were more similar when measured in situ under natural concentrations (Fig. 5). In situ diets were inferred by comparing the water In and Ex by the sponges and revealed that both species removed similar amounts of POC (measured as pico- and nanoplankton) from the ambient water (Fig. 5a). However, this POC removal represented only a fraction of the total net organic carbon removed by both sponges, as the majority of their natural diet comprised DOC. The HMA sponge *A. aerophoba* was more efficient at removing DOC, removing 12 µmol/L compared with 5 µmol/L for *D. avara* (Fig. 5a); but, since *D. avara* had a significantly higher pumping rate (*P*_perm_ < 0.05; Fig. 5b), total DOC and POC uptake rates were actually higher for the LMA sponge (Fig. 5c). In total, DOC accounted for 92.4 ± 4.4% and 87.3 ± 2.2% of the heterotrophic carbon consumed by the HMA and LMA sponge, respectively (Fig. 5c).

Using the host and symbionts percent contributions estimated for uptake of algal DOM—the DOM source likely to be most representative of the in situ DOM pool—we calculate that symbiont microbes account for 60 ± 3 % of the total heterotrophic C assimilated by the HMA sponge *A. aerophoba*. In contrast, sponge cells account for majority of C assimilated by the LMA sponge *D. avara*, with symbiont microbes accounting for <0.5% (Fig. 5d).

## Discussion

Studies of marine symbioses have mainly focussed on associations with photo- or chemoautotrophic microorganisms that play crucial roles in supplying their hosts with carbon in food-limited ocean environments, such as the coral-*Symbiodinium* holobiont on coral reefs [49] and bathymodiolin mussels on deep-sea hydrothermal vents [50]. Despite the fact that these autotrophic associations represent only a fraction of the diverse host–microbe associations occurring in marine animals [51], examples of microbial symbionts contributing majorly to heterotrophic nutrition are rare [52]. Here, we show that in one of the earliest known animal-microbe associations, microbes can make a substantial contribution to the host diet through efficient assimilation of the largest, and to many animals inaccessible, heterotrophic food source in the oceans: DOM.

Contrary to prevailing opinion that sponge DOM uptake is exclusively mediated by symbiotic microbes [6, 15, 21], our study supports recent findings that sponge cells are directly involved in DOM uptake [40]. By quantifying the contribution of host and symbiont cells to DOM uptake in sponges with differing abundances of microbial symbionts we show that the ratio between host versus symbiont processing of DOM changed depending on the abundance of symbionts, allowing both the high- and low-microbial abundance sponge to effectively exploit DOM. Sponges have been hypothesized to rely on DOM since the Ediacaran Period ~635 million years ago [53], and their plasticity in strategies to exploit DOM is likely a key factor in their long-term ecological success.

### DOM is assimilated by both host and symbiont cells

NanoSIMS visualization of distinct ^13^C and ^15^N enrichment hotspots concentrated exclusively in host choanocyte cells substantiates conclusions by Achlatis et al. [40] for the bioeroding sponge *Cliona orientalis* that the same cells responsible for the capture and phagocytosis of bacterial food [43–45] are also the primary host cells involved in the uptake of DOM. Together these findings corroborate studies showing high DOM turnover by sponges though rapid cell proliferation and turnover of choanocytes [54, 55]. Choanocyte cells are unique to *Porifera* and their involvement in DOM uptake likely explains the higher capacity for DOM uptake in sponges compared with other marine invertebrates. While the exact mechanisms by which DOM is taken up are not fully clear, DOM-fed choanocytes exhibit similar spatial enrichment patterns as for those fed with food bacteria, with ^13^C and ^15^N localized within vesicles (Fig. 3), supporting the proposal that DOM uptake by choanocyte cells likely occurs via pinocytosis [40]. Indeed, sponge choanocytes exhibit macropinocytotic activity [56] and high expression of genes involved in macropinocytosis [57, 58]; nevertheless, membrane transporters may also play a role as in other marine invertebrates [27, 57].

Sponge-associated microbes possess diverse transporters for the uptake of organic compounds [59–62], but their extracellular location in the inner sponge mesohyl requires that these compounds first pass through the epithelia of the either pinacoderm or choanoderm before it is accessible. Dissolved substances may be transported across the membranes of choanocytes or the endopinacocytes lining the incurrent canals of the host aquiferous system [57] or pass through leaky cell junctions to enter the mesophyll directly [44, 63]. Despite hosting distinct and diverse microbial communities (Fig. 1e, f), the majority of microbes in both the HMA and LMA sponge were enriched in ^13^C or ^15^N in the amino acids and algal DOM treatments (64–97%), indicating DOM utilization is a common trait in sponge-associated microbes. Indeed, diverse heterotrophic metabolic capabilities are a consistent feature in the genomes of sponge symbionts [59–62, 64–68] and widespread use of DOM by sponge-associated microbes, combined with high host activity, could explain inability to link sponge DOM uptake to specific microbial phyla [69]. Nevertheless, the DOM pool represents a diverse and heterogenous mixture of substances and differences in utilization of our three DOM sources suggest that, similar to DOM compartmentalization by free-living seawater microbes [70], there is likely to be metabolic specialization within the sponge microbiome for certain dissolved compounds [59–61].

Despite the fact that both host and microbial cells were active in DOM uptake, compared with host cells, microbial sponge symbionts more efficiently utilized the full range of dissolved compounds measured, particularly glucose and amino acids, which translated into significantly higher uptake rates of these compounds in the HMA compared with the LMA sponge. DOM is a heterogeneous mixture of substances operationally defined as all organic matter that can pass a “fine” filter (typically <0.7 µm), including particles and colloids smaller than the mesh size [71]. It has been proposed that sponge cells may utilize this colloidal fraction of DOM, while the associated microbes consume the truly dissolved material [15, 39]. Although incorporation of amino acids into choanocytes demonstrates clear uptake of true low-molecular weight DOM by host cells (Fig. 3), it is possible that their higher uptake of the algal DOM might be due to the colloidal content in this food source. The exact composition of algal DOM is unknown but consists of the complex mixture of compounds released during algal cell lysis. By expanding host access to a wider variety of compounds the sponge microbiome provides an important function analogous to the gut microbiomes of higher animals [72]. Despite lacking organs, sponges essentially function as efficient uptake systems and indeed represent an early multicellular uptake system [1], suggesting functional similarities between the microbiomes of this ancient “gut” and higher animals [55].

### HMA and LMA sponges have different strategies for DOM uptake

**Fig. 6:**
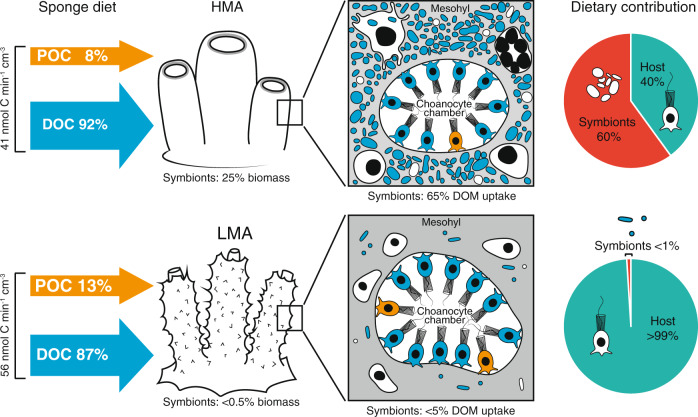
Conceptual diagram summarizing differences in DOM uptake strategies and symbiont contribution to heterotrophic diet in the HMA and LMA sponge. DOM is the primary carbon source for both the LMA and HMA sponge, accounting for ~90% of the in situ heterotrophic diet in both species. Both host sponge cells and bacterial symbionts are active in DOM assimilation, with the relative contributions differing based on the abundance of microbial symbionts. Higher symbiont biomass in the HMA sponge compared with that of the LMA sponge (25% vs. <0.5% of the total cellular biomass) leads to symbionts accounting for a larger contribution of the assimilated DOM in the HMA sponge than in LMA sponge (~65% vs. <5%). POM uptake accounts for about 10% of the diet in both sponges and is assimilated exclusively by the host sponge cells. Accordingly, the quantitative contribution of symbionts to the overall heterotrophic diet (DOC + POC) is ~60 and <1% for the HMA and the LMA sponge species, respectively.

DOM uptake in sponges is typically inferred from either SIP (e.g., [25, 37]) or in situ flux measurements (e.g., [22, 23]). Elevated food concentrations caused by the application of isotopically labeled substrates in SIP experiments may result in uptake rates that deviate from those under natural conditions. However, particularly when combined with techniques like NanoSIMS, fine details on processing can be captured that are overlooked when measuring net fluxes under natural conditions. Here, we combine both techniques, allowing us to quantitatively determine sponge feeding rates under natural in situ conditions while also disentangling the contribution of host and symbiont cells to nutrient acquisition at the single-cell level. We show that despite exhibiting significant differences in DOM uptake rates during ex situ isotope tracer experiments (Fig. 2), both the HMA and LMA sponge were well-adapted to taking up DOM in situ, with DOC accounting for ~90% of the natural diet of both species (Fig. 5). However, the role of microbial symbionts in mediating DOM uptake was strikingly different in the two sponge types. The HMA sponge relied heavily on its microbial symbionts for DOM uptake with microbes accounting for the majority (65–89%). By comparison, due to their low abundance, symbiont microbes made a very low contribution to DOM uptake in the LMA sponge (<5%) and instead DOM was taken up almost entirely by host cells (Fig. 6). Symbiotic microbes often provide their host with entirely novel functions (e.g., photosynthesis), but in this case the two sponges appear to have evolved two strategies—one largely microbial-mediated and the other host-driven—for accomplishing the same heterotrophic function. A widely acknowledged hypothesis for the HMA–LMA dichotomy is that HMA sponges have evolved to DOM-feeding, while LMA sponges solely use particulate food to meet their energy demands [6, 15], but our results, and earlier findings (see 5) indicate this is not the case.

HMA sponges have been considered better adapted for DOM uptake due to: (1) higher symbiont densities and (2) slower pumping rates which increase the residence time of water in the sponge aquiferous system and therefore the contact time for the cells to access DOM [6]. However, the relationship between HMA/LMA status and pumping rate has recently been questioned [7] and we found limited evidence that decreased pumping resulted in increased DOM uptake as we consistently found similar enrichments in host and symbiont cells of both species despite differences in pumping rate (Fig. 4). Moreover, while the HMA sponge was more efficient at taking up DOC in situ, removing 12 µmol C L^−1^ compared with 5 µmol C L^−1^ by the LMA sponge, the higher pumping rate in the LMA sponge actually resulted in an overall higher DOC uptake rate (Fig. 5b, c). These and recent findings suggest relationships between DOM uptake and microbial abundance/pumping rate are more complex [5, 73] and are further influenced by factors such as sponge growth form [5] as well as DOM quantity and composition [22, 37, 74, 75]. Microbes did appear to allow the HMA sponge to efficiently utilize a greater variety of dissolved compounds, which could contribute to trophic niche partitioning between HMA and LMA sponges [22, 76, 77]. Niche partitioning is an important factor in enabling co-existence of species and is thought to play a role in explaining the high densities of co-occurring HMA and LMA sponges [22, 76, 77]. DOC is by far the most abundant source of organic carbon in the ocean [12], and it appears that the two sponge types have evolved different strategies to exploit DOM, strongly suggesting that DOM utilization is a key factor in the long-term ecological success of marine sponges.

### Symbiont contributions to the heterotrophic sponge diet

Microbes not only accounted for the majority of DOM uptake in the HMA sponge, but also accounted for more than half of the total heterotrophic C assimilated by the HMA sponge. For some sponge species, chemo- and photosynthetic microbes are known to provide the sponge host with autotrophically fixed C [10, 42, 78–82], but here we provide quantitative data demonstrating that microbial sponge symbionts also contribute to the acquisition of heterotrophic C. We calculate that microbial symbionts are responsible for more than half (~60%) of the total heterotrophic C assimilated by the HMA sponge (Fig. 6). Although microbes were similarly active in the LMA sponge, due to their low numbers, their contribution to total C assimilation was overall much lower (<1%). Although we did not detect translocation of microbial-assimilated C and N to the sponge host, the 9-h timeframe may have been insufficient to detect potential nutrient transfer [78]. Translocation of low-molecular weight compounds [79, 83] and phagocytosis of microbes appear as the main potential mechanisms for translocation [21]. High rates of phagocytosis in the HMA sponge *Geodia barretti* are estimated to be sufficient to allow for a significant proportion of microbial-assimilated DOM to be transferred to the host [21], which is consistent with our observations of host cells engulfing symbionts in the HMA sponge. Moreover, even in LMA sponges, heterotrophic microbes likely contribute to host nutrition through the provision of essential vitamins and amino acids [61, 68, 84], similarly to the gut microbes of terrestrial animals [85, 86]. Although symbiont contributions to sponge diet are likely to vary across species, we show they can make a major contribution to heterotrophic nutrient acquisition by the sponge holobiont through the assimilation of DOM.

## Conclusions

We quantitatively demonstrate that both microbial symbionts and host cells are actively involved in DOM processing, enabling both HMA and LMA sponges to effectively utilize DOM. The relative contribution of symbionts differs along the HMA–LMA dichotomy due to variations in microbial biomass; while microbes accounted for the majority of DOM uptake in the HMA sponge, they made a minimal contribution in the LMA sponge, which instead relied on uptake by host choanocyte cells. A complex interplay between DOM quality and quantity, host physiology, and symbiont abundance is likely to influence DOM uptake in marine sponges. We show that, similar to autotrophic symbionts, heterotrophic symbionts can play an important role in nutrient acquisition in marine sponges—accounting for more than half of the total C assimilated by the HMA sponge. Further studies are needed to establish the exact mechanisms by which DOM is taken up, whether the host can regulate symbiont access to DOM, and the degree to which assimilated C and N is exchanged between host and symbionts. The fact that both types of marine sponge have evolved different strategies to capitalize on DOM, the largest reservoir of organic carbon in the ocean, underscores its importance to the sponge diet.

## Supplementary information

Supplemental Material
