# Supplementary material for: Heterotrophy in the earliest gut: a single-cell view of heterotrophic carbon and nitrogen assimilation in sponge-microbe symbioses
Source: ISME J. 2020 Jun 29;14(10):2554–67. doi: 10.1038/s41396-020-0706-3 (PMC7490408; doi:10.1038/s41396-020-0706-3)
Supplement: Supplementary file 1 — Supplemental Material [file 41396_2020_706_MOESM1_ESM.docx]

**Supplementary Information**

Heterotrophy in the earliest gut: A single-cell view of heterotrophic carbon and nitrogen assimilation in sponge-microbe symbioses

Laura Rix, Marta Ribes, Rafel Coma, Martin T. Jahn, Jasper M. de Goeij, Dick van Oevelen, Stéphane Escrig, Anders Meibom, Ute Hentschel

**Table of Contents**

Supplementary Materials and Methods………………………………………………………..2

Supplementary Results………………………………………………………………………...5

Supplementary Figures………………………………………………………………………...6

Figure S1………………………………………………………………………………6

Figure S2………………………………………………………………………………7

Supplementary Tables…………………………………………………………………………8

Table S1………………………………………………………………………………..8

Table S2………………………………………………………………………………..8

Table S3………………………………………………………………………………..9

Table S4………………………………………………………………………………..9

Table S5………………………………………………………………………………10

Table S6………………………………………………………………………………11

Table S7………………………………………………………………………………12

Table S8………………………………………………………………………………13

References……………………………………………………………………………………16

**Supplementary Materials and Methods**

**Microbial community analysis.** Microbial community composition was assessed by Illumina sequencing of the 16S sRNA taxonomic gene from 6 individuals of each sponge species collected in June 2017. DNA was extracted from frozen tissue (~0.25 g) using the DNeasy Power Soil Kit (Qiagen, Germany) as per the manufacturer’s instructions. DNA quantity and purity were measured using a NanoDrop spectrophotometer and gel electrophoresis after a polymerase chain reaction (PCR) with universal 16 rRNA gene primers. The V3 to V4 variable regions of the 16S rRNA gene were amplified using the primer pair 341F 5’-CCTACGGGAGGCAGCAG-3’ & 806R 5’-GGACTACHVGGGTWTCTAAT-3’ and sequencing was performed on a MiSeq platform (MiSeqFGx, Illumina). Sequences were processed using QIIME2 (version 2018.11) with default parameters. The DADA2 algorithm was applied on forward reads (truncated to 250nt) to generate Amplicon Sequence Variants (ASVs) which were phylogenetically classified based on the Silva 132 99% OTUs 16S database and unassigned sequences removed.

**Preparation of ^13^C- and ^15^N-labelled food sources for stable isotope pulse-chase experiments.** The glucose (99 atm% ^13^C) and algal-derived amino acid mixture (>97 atm% ^13^C and ^15^N) were commercially available from Cambridge Isotopes (Massachusetts, USA). The algal-DOM and bacteria were produced as follows. The diatom *Skeletonema costatum* was cultured axenically in F/2 media containing 2.1 nM NaHCO_3_ and 0.9 mM NaNO_3_ (98 atm% ^13^C and 99 atm% ^15^N, respectively; Cambridge Isotopes) at 24°C on a 12h:12h light/dark cycle. Once the diatoms had reached stationary growth phase (~2 weeks), they were centrifuged for 5 min at 1500 rpm, rinsed three times in artificial seawater, frozen at -80°C, and lyophilized. To extract DOM, cells lysis was induced by the addition of MQ followed by 5 min of vortexing and 20 min ultrasonication. The suspension was centrifuged at 4000 g for 5 min to pellet any remaining particulate material and the supernatant filtered to 0.2 µm. The extracted DOM was then lyophilized and stored at -80°C until use in experiments. The diatom DOM was supplemented with DOM produced from commercially available labelled cyanobacteria (98 atm% ^13^C and ^15^N; Cambridge Isotopes) extracted using the same method as the diatom DOM. This mixture was intended to replicate a more natural source of DOM as would be produced *in situ* by pelagic phytoplankton (1-3). Glucose and amino acids were selected as sugars and amino acids represent components of the DOM pool that are known to be highly labile to free-living heterotrophic microbes in the water column (1, 4-6)

To generate ^13^C and ^15^N labelled bacteria, a naturally community of seawater bacteria obtained from 5 ml of Baltic seawater was added to 1L of modified M63 media containing 0.02 mol glucose (50 atm% ^13^C, Cambridge Isotopes) and 0.01 mol ammonium chloride (99 atm% ^15^N, Cambridge Isotopes) and cultured in the dark at 28°C on a shaker. After 3 days, once the cells had reached stationary phase, samples were taken for cell counts and C/N elemental analysis and the bacteria were centrifuged at 4000 g for 5 min, rinsed with label-free ASW, and frozen at -20°C until use in experiments. The isotope ratios and C/N content of the algal DOM and bacteria were measured by EA-IRMS prior to use in experiment to determine the isotopic enrichment and total amounts of C and N added to experiments (Table S1).

**Stable isotope analysis and calculations of food uptake rates**. Bulk tissue samples were lyophilized, homogenized, and sub-samples weighed into silver (C) and tin (N) cups for stable isotope analysis of δ^13^C and δ^15^N. Samples for δ^13^C were decalcified with 0.4M HCl to obtain the organic carbon content. Separated cell fractions were lyophilized and weighed into tin cups for simultaneous δ^13^C and δ^15^N as test samples indicated that acidification was not required. Isotope ratios and C/N content were simultaneously measured using a Thermo FlashEA 1112 elemental analyser (EA) coupled to a Delta V isotope ratio mass spectrometer (IRMS). Carbon and nitrogen stable isotope ratios are expressed in standard delta notation as:

$\delta{}_{-}^{13}{C or \delta{}_{-}^{15}{N(}}‰)=\left( \frac{R_{sample}}{R_{ref}}-1 \right) \times1000$ (1)

where R is the ratio of ^13^C/^12^C or ^15^N/^14^N in the sample or reference material: Vienna Pee Dee Belemnite for C (R_ref_ = 0.01118) and atmospheric nitrogen for N (R_ref_ = 0.00368 N). Bulk tissue uptake rates were calculated as follows:

$F_{sample or bckgr}=\frac{{}_{-}^{13}C}{({}_{-}^{13}C+{}_{-}^{12}{C)}} or \frac{{}_{-}^{15}N}{({}_{-}^{15}N+{}_{-}^{14}{N)}}=\frac{R_{sample or bckgr}}{(R_{sample or bckgr}+1)}$ (1)

Where *F* is the fractional abundance of ^13^C or ^15^N in the samples was calculated as and:

$R_{sample or bckgr}=\left( \frac{\delta{}_{-}^{13}{C or \delta{}_{-}^{15}N}}{1000 +1} \right)\times R_{ref}$ (2)

Sample enrichments (*E*) was calculated as the excess fractional abundance (*F*) of ^13^C or ^15^N compared to control samples:

$E_{sample}=F_{sample}-F_{bckgr}$ (3)

Total ^13^C and ^15^N incorporation (*I)* was calculated by multiplying the excess fractional abundance (*E_sample_*) by the total C_org_ or N content (µmol) of the sample (*A*) divided by the fractional abundance of the different labelled food sources (*F*_source_):

$I=\frac{(E_{sample}\times A)}{E_{source}}$ (4)

Values for *E_source_* are presented in Table S1. Uptake rates (*Q*) were normalized to the sponge biomass (*B_sponge_*) measured as the total sponge C_org_ or N content (mmol) and the labelling incubation time (*t*):

$Q=\frac{I}{(B_{sponge}\times t)}$ (5)

*Q* is presented as µmol C or N incorporated per mmol C or N of sponge biomass per h (i.e. µmol C or N mmol C or N_sponge_^-1^ h^-1^; Fig. 2). All time points from the pulse-chase experiment are included in Figure 2.

**Separation of sponge and microbial cell fractions**. Methods for separation of sponge tissue into sponge and microbial cell fractions were adapted from Wehrl et al. 2007 (7) and Freeman et al. 2013 (8). Tissue samples were incubated in calcium and magnesium free artificial seawater with EDTA (CMFASW+EDTA) for 1 h at 4 °C and transferred to a clean 50 ml falcon tube filled with fresh ice-cold CMFASW+EDTA. All subsequent sampling steps were performed on ice or at 4 °C. Tissue samples were homogenized with a mortar and pestle to gently dissociate the tissue and passed through 100 µm Nitex mesh to remove any undissociated tissue. The filtrate was resuspended in 50 ml CMFASW+EDTA in clean 50 ml falcon tubes, vortexed for 5 min, and centrifuged to separate the sponge and microbial cells. For optimal purity, *A. aerophoba* was centrifuged at 770 g for 4 min and *D. avara* for 1100 g for 4 min. The supernatant containing the microbial cells was pipetted into a new falcon tube, discarding the last 5 ml to prevent contamination with the sponge pellet. The pellet containing the sponge cells was resuspended in fresh CMFASW+EDTA, vortexed again for 5 mins, and centrifuged for 4 mins this time at 520 g for *A. aerophoba* and 770 g for *D. avara* to remove any final microbes. The supernatant was poured off and the pellet rinsed one more time in CMFASW and centrifuged at the first centrifugation speeds for 4 min. The initial supernatant containing the microbial cell fraction was re-centrifuged at the initial speeds until no further sponge cells were pelleted and the resulting supernatant containing the microbial cells was centrifuged at 2800 g for 20 min followed by two rinsing steps; once in CMFASW+EDTA and once in CMFASW. The final sponge and microbial cell pellets were resuspended in 1ml CMFASW, transferred to 1.5ml Eppendorf tubes, and centrifuged at 1000 g for 2 min and 7000 g for 5 min, respectively. The remaining supernatant was removed and the pellet frozen at -80 °C.

To determine the number of sponge and microbial cells present in the initial homogenate and to test the purity of the sponge and microbial cell fractions, samples were taken from the initial 100-µm filtered homogenate and the purified sponge and microbial cell fractions (N = 176 in total). These samples were fixed with 1% paraformaldehyde, vortexed, fixed at room temperature for 10 mins, and frozen at -80 °C until cell counts were performed. For the cell counts, the thawed samples were stained with DAPI (1µg ml^-1^) and counted using a C-Chip Neubauer improved hemocytometer (Carl Roth, Germany) on an Axio Observer.Z1 microscope equipped with AxioCam 506 and Zen 2 version 2.0.0.0 software (Carl Zeiss Microscopy GmbH, Göttingen, Germany) at 40x magnification. Combined brightfield and DAPI signals were used to quantify stained cells according to the manufacturer instructions (C-Chip Neubauer improved, Carl Roth, Germany). All 3-h controls and samples were counted (N = 56). Purity of cell fractions was >99 % for the microbial cell fractions and >85 % for the sponge cell fractions (Table S2).

***In situ* InEx VacuSIP sample collection and analysis**. Samples for DOC (10 ml) were filtered *in situ* to 0.7 µm using in-line stainless steel filter holders with pre-combusted GF/F filters directly into pre-cleaned 40 ml EPA vials. Samples were brought to the surface and immediately fixed with 25% orthophosphoric acid (Ultrapure Sigma 79617) and stored in the dark at 4 °C until analysis by high-temperature combustion on Shimadzu a TOC analyser later that day. For POC, unfiltered triplicate samples for picoplankton (2 ml) were fixed with 1% paraformaldehyde and 0.5% electron microscopy grade glutaraldehyde, frozen in liquid nitrogen, and stored at -80 °C until analysis by flow cytometry. Although some sponges also consume detritus, this was not measured due to the long sampling time required (>8 h) and because detritus is not an important food source for *D. avara* (9). Phytoplankton (*Prochlorococcus* sp.*, Synechococcus* sp.*,* and photosynthetic pico- and nanoeukaryotes) and heterotrophic bacterioplankton were enumerated on a Becton-Dickinson FACSCalibur flow cytometer (488 nm excitation blue laser) as per (10, 11). Carbon contents of the different cell types used to calculate POC fluxes were estimated based on the literature conversion factors in (10).

**Supplementary Results**

### Translocation of C and N between symbiont and host cells. The separated cell fractions showed no evidence for translocation of microbial-assimilated C and N to the sponge host within the 9-h timeframe of the experiment as there was no significant increase in δ^13^C or δ^15^N values in the sponge cells between the end of the 3-h isotopic pulse and 9-h chase period (Fig. S1). However, we did observe host cells engulfing symbionts cells (Fig. S2), suggesting a potential mechanism by which C and N could be transferred from the symbionts to host cells. There was a small but significant increase in δ^13^C and/or δ^15^N values in the microbial cell fractions of the HMA sponge between the pulse and chase periods in the glucose and amino acids treatments (Fig. S1), which suggests transfer of C and N from host to symbiont cells. Interestingly, we also detected ^13^C and ^15^N enrichment in symbiont cells in the bacteria treatment in the NanoSIMS data after only 3 h (Fig. 4D, K). Since food bacteria are phagocytosed by host choanocyte cells and either digested or transferred to mesohyl cells for digestion, this demonstrates that host assimilated food bacteria were rapidly digested and the processed C and N waste products are rapidly recycled by microbial symbionts residing in the sponge mesohyl.

**Supplementary Figures**

**Figure S1.** Above background enrichment of ^13^C (A-C) and ^15^N (D-E) in the separated host sponge cells and symbiont microbial cell fractions for the three dissolved food sources: glucose (A), amino acids (B, D), and algal DOM (C, E) over the 6 time points in the pulse-chase experiments (0, 0.5, 1, 3, 6, 9h) in the HMA sponge *Aplysina aerophoba* and LMA sponge *Dysidea avara*. The horizontal grey line denotes the background levels of ^13^C and ^15^N in the control samples. The vertical grey dashed line at 3h represents the end of the labelling pulse phase and start of the chase phase. Note the different y-axis scale in D. Markers denote significant enrichment compared to controls for HMA symbiont microbes*, HMA host sponge cells^†^, LMA symbiont microbes^‡^, and LMA host sponge cells^§^. Letters denote significant differences between the final pulse time point (3h) and the two chase time points (6h and 9h) within the individual cell fractions. Differences were considered significant at P_(perm)_ < 0.05 based on PERMANOVA tests.


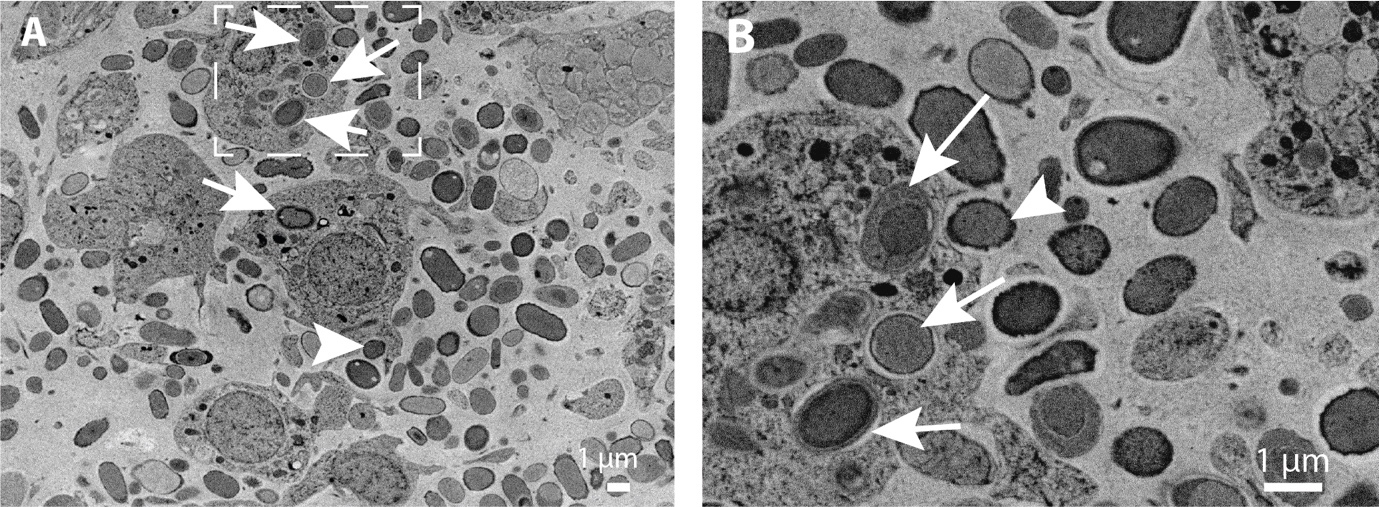


**Figure S2.** Phagocytosis of symbiont bacteria by host sponge cells in the HMA sponge *Aplysina aerophoba*. (A) Host cells containing multiple intracellular symbiont bacteria. (B) Close up of the area in the dashed outline in (A). Arrows point to intracellular symbiont bacteria that have already been phagocytosed while arrow heads indicate symbiont bacteria currently in the process of being engulfed by host cells.

**Supplementary Tables**:

**Table S1**. Summary of the isotopic enrichment of ^13^C and ^15^N (atom %), concentration (µM) of carbon (C) and nitrogen (N), and total amounts (µmol) of the four food sources added to the stable isotope probing experiments.

|  | Carbon | | | Nitrogen | | |
| --- | --- | --- | --- | --- | --- | --- |
| Food source | Concentration  (µM) | Total (µmol) | Atm^13^C% | Concentration (µM) | Total (µmol) | Atm^15^N% |
| Glucose | 81.8 | 491 | 99 | - | - | - |
| Amino acids | 79.7 | 478 | 99 | 20.0 | 120 | 99 |
| Algal DOM | 77.6 | 466 | 36 | 8.2 | 49 | 43 |
| Bacteria | 80 | 480 | 50 | 19.8 | 118 | 99 |

**Table S2.** Two-way PERMANOVA testing for significant effects of treatment and time point on bulk tissue C or N assimilation rates in the two sponge species: the high-microbial abundance (HMA) sponge *Aplysina aerophoba* and the low-microbial abundance (LMA) sponge *Dysidea avara*. Pseudo-F = pseudo F-statistic, *P*_(perm)_ = permutational *P*-value. Unique permutations = 9999. Values in bold are statistically significant at the level of *P*_(perm)_ < 0.05.

|  |  | Carbon | | | Nitrogen | | |
| --- | --- | --- | --- | --- | --- | --- | --- |
|  | Factor | df | Pseudo-F | *P*_(perm)_ | df | Pseudo-F | *P*_(perm)_ |
| HMA | Treatment | 3 | 59.675 | **0.0001** | 2 | 78.255 | **0.0001** |
|  | Time | 4 | 2.0175 | 0.1872 | 4 | 2.9865 | 0.1278 |
|  | Tr x Ti | 12 | 2.1954 | 0.0874 | 8 | 1.9775 | 0.204 |
|  | Residuals | 55 |  |  | 40 |  |  |
| LMA | Treatment | 3 | 45.85 | **0.0001** | 2 | 40.186 | **0.0001** |
|  | Time | 4 | 2.8033 | 0.0653 | 4 | 1.6969 | 0.1869 |
|  | Tr x Ti | 12 | 2.1039 | 0.0799 | 8 | 1.45 | 0.2214 |
|  | Residuals | 55 |  |  | 40 |  |  |

**Table S3.** Summary of the percentage of host sponge cells and symbiont microbes present pre- and post-separation into cell fractions. Pre-separation values represent the initial ratio of sponge and microbial cells in the sponge tissue. Post-separation values show purity of the sponge and microbial cell fractions after separation.

|  | HMA | | LMA | | |
| --- | --- | --- | --- | --- | --- |
|  | Microbes (%) | Sponge cells (%) | Microbes (%) | Sponge cells (%) |  |
| Pre-separation |  |  |  |  |  |
| Total cells | 98.7 | 1.3 | 39.5 | 60.5 |  |
| Post-separation |  |  |  |  |  |
| Sponge fraction | 14.8 | 85.2 | 9.6 | 90.4 |  |
| Microbial fraction | 99.7 | 0.3 | 99.5 | 0.5 |  |

**Table S4**. Comparison of the percent contribution of host sponge cells and symbiont microbes to the uptake of the different DOM sources using the cell fraction stable isotope data (CF) and NanoSIMS stable isotope data (NS).

|  | HMA | | | | LMA | | | |
| --- | --- | --- | --- | --- | --- | --- | --- | --- |
|  | Symbiont (%) | | Host (%) | | Symbiont (%) | | Host (%) | |
| DOM source | CF | NS | CF | NS | CF | NS | CF | NS |
| C |  |  |  |  |  |  |  |  |
| Glucose | 86.7 | 85.2 | 13.3 | 14.8 | 4.1 | 1.0 | 95.9 | 99.0 |
| Amino acids | 85.0 | 60.6 | 15.0 | 39.4 | 1.8 | 1.1 | 98.2 | 98.9 |
| Algal DOM | 65.4 | 61.9 | 34.6 | 38.1 | 0.4 | 1.4 | 99.6 | 98.6 |
| N |  |  |  |  |  |  |  |  |
| Glucose | - | - | - | - | - | - | - |  |
| Amino acids | 86.1 | 88.6 | 13.9 | 11.4 | 5.0 | 2.4 | 95.0 | 97.6 |
| Algal DOM | 71.5 | 85.8 | 28.5 | 14.2 | 1.4 | 2.2 | 98.6 | 97.8 |

**Table S5.** Summary of NanoSIMS data collection showing the number of replicate specimens per treatment per time point for each of the two sponge species (the HMA sponge *A. aerophoba* and LMA sponge *D. avara*), the number of raster regions imaged, and the number of Regions of Interest (ROIS) measured for each ROI type.

| Species | Treatment | Timepoint | | | Replicates | | Raster regions | | ROI | | N | |
| --- | --- | --- | --- | --- | --- | --- | --- | --- | --- | --- | --- | --- |
| HMA | Control | 3h | | | 2 | | 6 | | Choanocyte | | 39 | |
|  |  |  | | |  | |  | | Mesohyl | | 21 | |
|  |  |  | | |  | |  | | Bacteria | | 101 | |
|  | Glucose | 3h | | | 1 | | 4 | | Choanocyte | | 23 | |
|  |  |  | | |  | |  | | Mesohyl | | 22 | |
|  |  |  | | |  | |  | | Bacteria | | 209 | |
|  | Amino acids | 3h | | | 2 | | 4 | | Choanocyte | | 30 | |
|  |  |  | | |  | |  | | Hotspot | | 18 | |
|  |  |  | | |  | |  | | Mesohyl | | 20 | |
|  |  |  | | |  | |  | | Bacteria | | 552 | |
|  | Algal DOM | 3h | | | 2 | | 5 | | Choanocyte | | 41 | |
|  |  |  | | |  | |  | | Hotspot | | 27 | |
|  |  |  | | |  | |  | | Mesohyl | | 25 | |
|  |  |  | | |  | |  | | Bacteria | | 821 | |
|  | Bacteria | 3h | | | 2 | | 8 | | Choanocyte | | 93 | |
|  |  |  | | |  | |  | | Hotspot | | 124 | |
|  |  |  | | |  | |  | | Mesohyl | | 35 | |
|  |  |  | | |  | |  | | Bacteria | | 1210 | |
| LMA | Control | 3h | | | 2 | | 8 | | Choanocyte | | 49 | |
|  |  |  | | |  | |  | | Mesohyl | | 20 | |
|  |  |  | | |  | |  | | Bacteria | | 91 | |
|  | Glucose | 3h | | | 1 | | 5 | | Choanocyte | | 63 | |
|  |  |  | | |  | |  | | Mesohyl | | 17 | |
|  |  |  | | |  | |  | | Bacteria | | 38 | |
|  | Amino acids | 3h | | | 2 | | 5 | | Choanocyte | | 46 | |
|  |  |  | | |  | |  | | Hotspot | | 15 | |
|  |  |  | | |  | |  | | Mesohyl | | 20 | |
|  |  |  | | |  | |  | | Bacteria | | 49 | |
|  | Algal DOM | 3h | | | 2 | | 8 | | Choanocyte | | 85 | |
|  |  |  | | |  | |  | | Hotspot | | 111 | |
|  |  |  | | |  | |  | | Mesohyl | | 18 | |
|  |  |  | | |  | |  | | Bacteria | | 38 | |
|  | Bacteria | 3h | | | 2 | | 6 | | Choanocyte | | 65 | |
|  |  |  | | |  | |  | | Hotspot | | 205 | |
|  |  |  | | |  | |  | | Mesohyl | | 31 | |
|  |  |  | | |  | |  | | Bacteria | | 41 | |
|  |  | | **Total** | **18** | | **59** | |  | | **4354** | |  |

**Table S6**. Pairwise comparisons showing significant differences in bulk C or N assimilation between the four treatments (food sources) for each species: the high-microbial abundance (HMA) sponge *Aplysina aerophoba* and the low-microbial abundance (LMA) sponge *Dysidea avara*. Glu = glucose, AA = amino acids, DOM = algal DOM, Bact = bacteria, *t* = pseudo *t*-statistic, *P*_(perm)_ = permutational *P*-value. Unique permutations = 9999. Values in bold are statistically significant at the level of *P*_(perm)_ < 0.05.

|  |  | C | | N | |
| --- | --- | --- | --- | --- | --- |
|  | Group | *t* | *P*_(perm)_ | *t* | *P*_(perm)_ |
| HMA | Glu, AA | 14.354 | **0.0001** | - | - |
|  | Glu, DOM | 4.7732 | **0.0001** | - | - |
|  | Glu, Bact | 2.3957 | **0.0445** | - | - |
|  | AA, DOM | 6.9893 | **0.0001** | 10.682 | **0.0001** |
|  | AA, Bact | 8.4741 | **0.0001** | 9.1876 | **0.0001** |
|  | DOM, Bact | 2.0145 | 0.0787 | 1.5766 | 0.2058 |
| LMA | Glu, AA | 6.9306 | **0.0001** | - | - |
|  | Glu, DOM | 20.877 | **0.0001** | - | - |
|  | Glu, Bact | 6.9816 | **0.0001** | - | - |
|  | AA, DOM | 6.9074 | **0.0001** | 5.5304 | **0.0001** |
|  | AA, Bact | 4.7496 | **0.0001** | 6.9298 | **0.0001** |
|  | DOM, Bact | 4.6372 | **0.0001** | 5.7624 | **0.0001** |

**Table S7.** Three-way PERMANOVA testing for significant differences in bulk C or N assimilation between sponge species for each treatment (food source) with time point as a factor. Pairwise comparisons show significant differences between the high-microbial abundance (HMA) sponge *Aplysina aerophoba* and the low-microbial abundance (LMA) sponge *Dysidea avara* for each treatment as time point was not found to be a significant factor. df = degrees of freedom, Pseudo-F = Pseudo-F statistic, *P*_(perm)_ = permutational *P*-value. Unique permutations = 9999. Values in bold are statistically significant at the level of *P*_(perm)_ < 0.05.

|  | Main PERMANOVA test | | | | Pairwise test (Sp x Tr) | | | | |
| --- | --- | --- | --- | --- | --- | --- | --- | --- | --- |
| C | Factor | df | Pseudo-F | *P*_(perm)_ | | Tr level | Groups | *t* | *P*_(perm)_ |
|  | Species | 1 | 3.9387 | 0.1733 | | Glu | HMA vs LMA | 26.18 | **0.0001** |
|  | Treatment | 2 | 50.548 | **0.0001** | | AA | HMA vs LMA | 16.932 | **0.0001** |
|  | Time | 4 | 3.6776 | 0.2233 | | DOM | HMA vs LMA | 1.2394 | **0.0569** |
|  | Sp x Tr | 2 | 47.8 | **0.0001** | | Bact | HMA vs LMA | 5.6166 | **0.0001** |
|  | Sp x Ti | 4 | 1.4826 | 0.282 | |  |  |  |  |
|  | Tr x Ti | 8 | 2.6813 | 0.0519 | |  |  |  |  |
|  | Sp x Tr x Ti | 8 | 1.6739 | 0.1741 | |  |  |  |  |
|  | Residuals | 80 |  |  | |  |  |  |  |
| N | Species | 1 | 1.1584 | 0.3432 | | AA | HMA vs LMA | 15.536 | **0.0001** |
|  | Treatment | 2 | 42.459 | **0.0001** | | DOM | HMA vs LMA | 1.7639 | 0.0913 |
|  | Time | 4 | 2.1673 | 0.1883 | | Bact | HMA vs LMA | 5.1926 | **0.0006** |
|  | Sp x Tr | 2 | 40.192 | **0.0001** | |  |  |  |  |
|  | Sp x Ti | 4 | 1.3036 | 0.3538 | |  |  |  |  |
|  | Tr x Ti | 8 | 1.3861 | 0.3216 | |  |  |  |  |
|  | Sp x Tr x Ti | 8 | 1.5455 | 0.2723 | |  |  |  |  |
|  | Residuals | 80 |  |  | |  |  |  |  |

**Table S8.** NanoSIMS data statistical summary: two-way PERMANOVA testing for significant differences between sponge species and ROI type for each food source. Choanocytes (C), choanocyte enrichment hotspots (HS), mesophyll cells (M), symbiont bacteria (B). The two sponge species are the high-microbial abundance (HMA) sponge *Aplysina aerophoba* and the low-microbial abundance (LMA) sponge *Dysidea avara*. df = degrees of freedom, Pseudo-F = Pseudo-F statistic, *P*_(perm)_ = permutational *P* value. Unique permutations = 9999. Values in bold are statistically significant at the level of *P*_(perm)_ < 0.05.

|  | Main PERMANOVA test | | | | | | Pairwise tests | | | | |
| --- | --- | --- | --- | --- | --- | --- | --- | --- | --- | --- | --- |
|  | | Factor | df | Pseudo-F | *P*_(perm)_ | Level | | Groups | *t* | *P*_(perm)_ |  |
| Glucose | | Species | 1 | 0.0015282 | 0.9733 | ROI | | C vs M | 22.202 | **0.0001** |  |
| ^13^C | | ROI | 2 | 11.194 | **0.0006** |  | | C vs B | 3.3318 | **0.0019** |  |
|  | | Sp x ROI | 2 | 0.8529 | 0.4233 |  | | M vs B | 3.8178 | **0.0009** |  |
|  | | Residuals | 77 |  |  | LMA | | C vs M | 21.086 | **0.0078** |  |
|  | |  |  |  |  |  | | C vs B | 1.2881 | 0.1449 |  |
|  | |  |  |  |  |  | | M vs B | 2.0605 | **0.0106** |  |
|  | |  |  |  |  | HMA | | C vs M | - | - |  |
|  | |  |  |  |  |  | | C vs B | 3.8553 | **0.0011** |  |
|  | |  |  |  |  |  | | M vs B | 3.5184 | **0.0031** |  |
|  | |  |  |  |  | C | | HMA vs LMA | 23.359 | **0.0029** |  |
|  | |  |  |  |  | M | | HMA vs LMA | - | - |  |
|  | |  |  |  |  | B | | HMA vs LMA | 0.98613 | 0.3315 |  |
| Amino acid | | Species | 1 | 0.27255 | 0.5934 | ROI | | C vs Hs | 4.8505 | **0.0003** |  |
| ^13^C | | ROI | 3 | 34.202 | **0.0001** |  | | C vs M | 7.6606 | **0.0001** |  |
|  | | Sp x ROI | 3 | 6.218 | **0.003** |  | | C vs B | 7.0015 | **0.0001** |  |
|  | | Residuals | 314 |  |  |  | | Hs vs M | 5.8474 | **0.0001** |  |
|  | |  |  |  |  |  | | Hs vs B | 0.91767 | 0.3401 |  |
|  | |  |  |  |  |  | | M vs B | 8.6523 | **0.0001** |  |
|  | |  |  |  |  | LMA | | C vs Hs | 4.1378 | **0.0015** |  |
|  | |  |  |  |  |  | | C vs M | 5.1599 | **0.0005** |  |
|  | |  |  |  |  |  | | C vs B | 14.596 | **0.0001** |  |
|  | |  |  |  |  |  | | Hs vs M | 5.8513 | **0.0075** |  |
|  | |  |  |  |  |  | | Hs vs B | 4.4337 | **0.0154** |  |
|  | |  |  |  |  |  | | M vs B | 22.713 | **0.0086** |  |
|  | |  |  |  |  | HMA | | C vs Hs | 3.1474 | **0.0073** |  |
|  | |  |  |  |  |  | | C vs M | 10.964 | **0.0076** |  |
|  | |  |  |  |  |  | | C vs B | 3.5855 | **0.0056** |  |
|  | |  |  |  |  |  | | Hs vs M | 4.0572 | **0.0071** |  |
|  | |  |  |  |  |  | | Hs vs B | 2.8427 | **0.0146** |  |
|  | |  |  |  |  |  | | M vs B | 5.3765 | **0.0004** |  |
|  | |  |  |  |  | C | | HMA vs LMA | 0.75278 | 0.5632 |  |
|  | |  |  |  |  | Hs | | HMA vs LMA | 1.3968 | 0.2059 |  |
|  | |  |  |  |  | M | | HMA vs LMA | - | - |  |
|  | |  |  |  |  | B | | HMA vs LMA | 3.953 | **0.0027** |  |
| Amino acid | | Species | 1 | 0.63734 | 0.4284 | ROI | | C vs Hs | 3.2812 | **0.004** |  |
| ^15^N | | ROI | 3 | 22.292 | **0.0001** |  | | C vs M | 0.88304 | 0.5099 |  |
|  | | Sp x ROI | 3 | 0.86369 | 0.4468 |  | | C vs B | 3.6833 | **0.0012** |  |
|  | | Residuals | 606 |  |  |  | | Hs vs M | 4.8653 | **0.0001** |  |
|  | |  |  |  |  |  | | Hs vs B | 5.1322 | **0.0001** |  |
|  | |  |  |  |  |  | | M vs B | 5.2261 | **0.0001** |  |
|  | |  |  |  |  | LMA | | C vs Hs | 1.8613 | 0.0915 |  |
|  | |  |  |  |  |  | | C vs M | 1.0104 | 0.3355 |  |
|  | |  |  |  |  |  | | C vs B | 1.4471 | 0.1536 |  |
|  | |  |  |  |  |  | | Hs vs M | 3.4252 | **0.0068** |  |
|  | |  |  |  |  |  | | Hs vs B | 3.7597 | **0.0031** |  |
|  | |  |  |  |  |  | | M vs B | 5.3473 | **0.0001** |  |
|  | |  |  |  |  | HMA | | C vs Hs | 3.1384 | **0.0112** |  |
|  | |  |  |  |  |  | | C vs M | 0.041518 | 0.9467 |  |
|  | |  |  |  |  |  | | C vs B | 3.4082 | **0.0035** |  |
|  | |  |  |  |  |  | | Hs vs M | 3.4688 | **0.0055** |  |
|  | |  |  |  |  |  | | Hs vs B | 3.7816 | **0.0008** |  |
|  | |  |  |  |  |  | | M vs B | 3.74 | **0.001** |  |
|  | |  |  |  |  | C | | HMA vs LMA | 0.83059 | 0.7067 |  |
|  | |  |  |  |  | Hs | | HMA vs LMA | 0.15726 | 0.8811 |  |
|  | |  |  |  |  | M | | HMA vs LMA | 4.7406 | **0.005** |  |
|  | |  |  |  |  | B | | HMA vs LMA | 0.30492 | 0.7607 |  |
| Algal DOM | | Species | 1 | 1.8437 | 0.1644 | ROI | | C vs Hs | 2.9588 | 0.0049 |  |
| ^13^C | | ROI | 3 | 9.8623 | **0.0014** |  | | C vs M | 5.1371 | **0.0001** |  |
|  | | Sp x ROI | 3 | 0.3673 | 0.6832 |  | | C vs B | 4.1758 | **0.006** |  |
|  | | Residuals | 381 |  |  |  | | Hs vs M | 2.9165 | **0.0072** |  |
|  | |  |  |  |  |  | | Hs vs B | 0.36729 | 0.624 |  |
|  | |  |  |  |  |  | | M vs B | 4.6291 | **0.0015** |  |
|  | |  |  |  |  | LMA | | C vs Hs | 2.9504 | **0.0066** |  |
|  | |  |  |  |  |  | | C vs M | 3.4404 | **0.0057** |  |
|  | |  |  |  |  |  | | C vs B | 6.7221 | **0.0006** |  |
|  | |  |  |  |  |  | | Hs vs M | 2.5666 | **0.0231** |  |
|  | |  |  |  |  |  | | Hs vs B | 0.04764 | 0.9628 |  |
|  | |  |  |  |  |  | | M vs B | 10.374 | **0.0088** |  |
|  | |  |  |  |  | HMA | | C vs Hs | 3.5786 | **0.0002** |  |
|  | |  |  |  |  |  | | C vs M | 36.482 | **0.0004** |  |
|  | |  |  |  |  |  | | C vs B | 3.0435 | **0.0198** |  |
|  | |  |  |  |  |  | | Hs vs M | 3.1615 | **0.0054** |  |
|  | |  |  |  |  |  | | Hs vs B | 1.0413 | **0.1625** |  |
|  | |  |  |  |  |  | | M vs B | 3.0177 | **0.0276** |  |
|  | |  |  |  |  | C | | HMA vs LMA | 1.9564 | **0.0443** |  |
|  | |  |  |  |  | Hs | | HMA vs LMA | 0.67273 | 0.5012 |  |
|  | |  |  |  |  | M | | HMA vs LMA | - | - |  |
|  | |  |  |  |  | B | | HMA vs LMA | 1.9162 | 0.0567 |  |
| Algal DOM | | Species | 1 | 8.6959 | **0.0229** | ROI | | C vs Hs | 3.5654 | **0.0027** |  |
| ^15^N | | ROI | 3 | 19.599 | **0.0005** |  | | C vs M | 0.8279 | 0.368 |  |
|  | | Sp x ROI | 3 | 2.9453 | 0.0799 |  | | C vs B | 10.014 | **0.0001** |  |
|  | | Residuals | 946 |  |  |  | | Hs vs M | 1.9282 | 0.0583 |  |
|  | |  |  |  |  |  | | Hs vs B | 5.5602 | **0.0014** |  |
|  | |  |  |  |  |  | | M vs B | 6.2162 | **0.0001** |  |
|  | |  |  |  |  | LMA | | C vs Hs | 8.2617 | **0.0001** |  |
|  | |  |  |  |  |  | | C vs M | 0.9054 | 0.1859 |  |
|  | |  |  |  |  |  | | C vs B | 10.774 | **0.0001** |  |
|  | |  |  |  |  |  | | Hs vs M | 1.9019 | 0.0628 |  |
|  | |  |  |  |  |  | | Hs vs B | 3.3729 | **0.0016** |  |
|  | |  |  |  |  |  | | M vs B | 3.4919 | **0.0016** |  |
|  | |  |  |  |  | HMA | | C vs Hs | 14.052 | **0.0001** |  |
|  | |  |  |  |  |  | | C vs M | 1.7869 | 0.0955 |  |
|  | |  |  |  |  |  | | C vs B | 3.0992 | **0.0043** |  |
|  | |  |  |  |  |  | | Hs vs M | 10.477 | **0.0005** |  |
|  | |  |  |  |  |  | | Hs vs B | 7.3941 | **0.0001** |  |
|  | |  |  |  |  |  | | M vs B | 2.4988 | **0.0194** |  |
|  | |  |  |  |  | C | | HMA vs LMA | 1.6042 | 0.0745 |  |
|  | |  |  |  |  | Hs | | HMA vs LMA | 1.4565 | 0.1044 |  |
|  | |  |  |  |  | M | | HMA vs LMA | 2.9369 | **0.0233** |  |
|  | |  |  |  |  | B | | HMA vs LMA | 12.998 | **0.0001** |  |
| Bacteria | | Species | 1 | 0.12152 | 0.717 | ROI | | C vs Hs | 12.257 | **0.0001** |  |
| ^13^C | | ROI | 3 | 93.318 | **0.0001** |  | | C vs M | 3.2804 | **0.0027** |  |
|  | | Sp x ROI | 3 | 0.19366 | 0.8909 |  | | C vs B | 5.2288 | **0.0004** |  |
|  | | Residuals | 619 |  |  |  | | Hs vs M | 5.0518 | **0.0001** |  |
|  | |  |  |  |  |  | | Hs vs B | 8.0309 | **0.0001** |  |
|  | |  |  |  |  |  | | M vs B | 9.4908 | **0.0001** |  |
|  | |  |  |  |  | LMA | | C vs Hs | 8.6105 | **0.0001** |  |
|  | |  |  |  |  |  | | C vs M | 2.6422 | **0.0108** |  |
|  | |  |  |  |  |  | | C vs B | 1.7319 | 0.0753 |  |
|  | |  |  |  |  |  | | Hs vs M | 3.6393 | **0.0019** |  |
|  | |  |  |  |  |  | | Hs vs B | 3.1759 | **0.0041** |  |
|  | |  |  |  |  |  | | M vs B | 4.347 | **0.0006** |  |
|  | |  |  |  |  | HMA | | C vs Hs | 8.8703 | **0.0001** |  |
|  | |  |  |  |  |  | | C vs M | 2.1601 | **0.0305** |  |
|  | |  |  |  |  |  | | C vs B | 10.624 | **0.0001** |  |
|  | |  |  |  |  |  | | Hs vs M | 3.5745 | **0.0017** |  |
|  | |  |  |  |  |  | | Hs vs B | 19.433 | **0.0001** |  |
|  | |  |  |  |  |  | | M vs B | 6.4084 | **0.0001** |  |
|  | |  |  |  |  | C | | HMA vs LMA | 1.7411 | 0.0838 |  |
|  | |  |  |  |  | Hs | | HMA vs LMA | 1.3754 | 0.1681 |  |
|  | |  |  |  |  | M | | HMA vs LMA | 6.8157 | **0.0001** |  |
|  | |  |  |  |  | B | | HMA vs LMA | 6.0217 | **0.0001** |  |
| Bacteria | | Species | 1 | 0.00345 | 0.95 | ROI | | C vs Hs | 11.113 | **0.0001** |  |
| ^15^N | | ROI | 3 | 214.68 | **0.0001** |  | | C vs M | 5.345 | **0.0001** |  |
|  | | Sp x ROI | 3 | 0.033318 | 0.9834 |  | | C vs B | 14.121 | **0.0001** |  |
|  | | Residuals | 1192 |  |  |  | | Hs vs M | 8.3331 | **0.0001** |  |
|  | |  |  |  |  |  | | Hs vs B | 19.375 | **0.0001** |  |
|  | |  |  |  |  |  | | M vs B | 14.962 | **0.0001** |  |
|  | |  |  |  |  | LMA | | C vs Hs | 7.3785 | **0.0001** |  |
|  | |  |  |  |  |  | | C vs M | 4.6551 | **0.0001** |  |
|  | |  |  |  |  |  | | C vs B | 4.8231 | **0.0001** |  |
|  | |  |  |  |  |  | | Hs vs M | 6.112 | **0.0001** |  |
|  | |  |  |  |  |  | | Hs vs B | 6.6221 | **0.0001** |  |
|  | |  |  |  |  |  | | M vs B | 5.9731 | **0.0001** |  |
|  | |  |  |  |  | HMA | | C vs Hs | 8.7391 | **0.0001** |  |
|  | |  |  |  |  |  | | C vs M | 3.3681 | **0.0012** |  |
|  | |  |  |  |  |  | | C vs B | 17.303 | **0.0001** |  |
|  | |  |  |  |  |  | | Hs vs M | 5.9368 | **0.0001** |  |
|  | |  |  |  |  |  | | Hs vs B | 30.548 | **0.0001** |  |
|  | |  |  |  |  |  | | M vs B | 4.2013 | **0.0005** |  |
|  | |  |  |  |  | C | | HMA vs LMA | 0.49718 | 0.6241 |  |
|  | |  |  |  |  | Hs | | HMA vs LMA | 0.028106 | 0.9772 |  |
|  | |  |  |  |  | M | | HMA vs LMA | 3.2169 | **0.0001** |  |
|  | |  |  |  |  | B | | HMA vs LMA | 34.725 | **0.0001** |  |

**References**

1. C. Zhang *et al.*, Evolving paradigms in biological carbon cycling in the ocean. *National Science Review* **5**, 481-499 (2018).

2. D. C. O. Thornton, Dissolved organic matter (DOM) release by phytoplankton in the contemporary and future ocean. *European Journal of Phycology* **49**, 20-46 (2014).

3. S. B. Baines, M. L. Pace, The production of dissolved organic matter by phytoplankton and its importance to bacteria: Patterns across marine and freshwater systems. *Limnology and Oceanography* **36**, 1078-1090 (1991).

4. R. Benner, R. M. W. Amon, The Size-Reactivity Continuum of Major Bioelements in the Ocean. *Annual Review of Marine Science* **7**, 185-205 (2015).

5. R. S. Poretsky, S. Sun, X. Mou, M. A. Moran, Transporter genes expressed by coastal bacterioplankton in response to dissolved organic carbon. *Environmental Microbiology* **12**, 616-627 (2010).

6. K. Bergauer *et al.*, Organic matter processing by microbial communities throughout the Atlantic water column as revealed by metaproteomics. *Proceedings of the National Academy of Sciences* **115**, E400 (2018).

7. M. Wehrl, M. Steinert, U. Hentschel, Bacterial uptake by the marine sponge *Aplysina aerophoba*. *Microbial Ecology* **53**, 355-365 (2007).

8. C. J. Freeman, R. W. Thacker, D. M. Baker, M. L. Fogel, Quality or quantity: is nutrient transfer driven more by symbiont identity and productivity than by symbiont abundance? *Isme Journal* **7**, 1116-1125 (2013).

9. M. Ribes, R. Coma, J. M. Gili, Natural diet and grazing rate of the temperate sponge *Dysidea avara* (Demospongiae, Dendroceratida) throughout an annual cycle. *Marine Ecology Progress Series* **176**, 179-190 (1999).

10. T. Morganti, G. Yahel, M. Ribes, R. Coma, VacuSIP, an improved InEx method for in situ measurement of particulate and dissolved compounds processed by active suspension feeders. *JoVE* doi:10.3791/54221, e54221 (2016).

11. T. Morganti, R. Coma, G. Yahel, M. Ribes, Trophic niche separation that facilitates co-existence of high and low microbial abundance sponges is revealed by in situ study of carbon and nitrogen fluxes. *Limnology and Oceanography* **62**, 1963-1983 (2017).
